# ^13^C and ^15^N natural isotope abundance reflects breast cancer cell metabolism

**DOI:** 10.1038/srep34251

**Published:** 2016-09-28

**Authors:** Illa Tea, Estelle Martineau, Ingrid Antheaume, Julie Lalande, Caroline Mauve, Francoise Gilard, Sophie Barillé-Nion, Anneke C. Blackburn, Guillaume Tcherkez

**Affiliations:** 1 EBSI team, CEISAM, University of Nantes-CNRS UMR 6230, 2 rue de la Houssinière BP 92208, F-44322 Nantes, France; 2 Cancer Metabolism and Genetics Group, The John Curtin School of Medical Research, The Australian National University, Canberra 2601 ACT, Australia; 3 Spectromaitrise, CAPACITÉS SAS, 26 Bd Vincent Gâche, 44200 Nantes, France; 4 Plateforme Métabolisme-Métabolome, Bâtiment 630 IPS2, Université Paris-Sud, 91405 Orsay cedex, France; 5 CRCNA, UMR INSERM U892/CNRS 6299/Université de Nantes, Team 8 «Cell survival and tumor escape in breast cancers», Institut de Recherche en Santé de l’Université de Nantes, 8 quai Moncousu, BP 70721, 44 007 Nantes cedex 1, France; 6 Research School of Biology, The Australian National University, Canberra 2601 ACT, Australia

## Abstract

Breast cancer is the most common cancer in women worldwide. Despite the information provided by anatomopathological assessment and molecular markers (such as receptor expression ER, PR, HER2), breast cancer therapies and prognostics depend on the metabolic properties of tumor cells. However, metabolomics have not provided a robust and congruent biomarker yet, likely because individual metabolite contents are insufficient to encapsulate all of the alterations in metabolic fluxes. Here, we took advantage of natural ^13^C and ^15^N isotope abundance to show there are isotopic differences between healthy and cancer biopsy tissues or between healthy and malignant cultured cell lines. Isotope mass balance further suggests that these differences are mostly related to lipid metabolism, anaplerosis and urea cycle, three pathways known to be impacted in malignant cells. Our results demonstrate that the isotope signature is a good descriptor of metabolism since it integrates modifications in C partitioning and N excretion altogether. Our present study is thus a starting point to possible clinical applications such as patient screening and biopsy characterization in every cancer that is associated with metabolic changes.

Medical applications of stable isotopes are now widespread, like the well-known ^13^C-urea breath assay for ulcer detection^1^. This takes advantage of ^13^C-labelling and thus usually neglects differences in reaction rates between isotopic forms, because the isotopic signal used for diagnosing is far above small natural variations in ^13^C. By contrast, the use of isotopes at natural abundance exploits such subtle differences (referred to as isotope effects) to identify bottlenecks in metabolic pathways (rate-limiting steps) or the contribution of multiple elemental sources (mass balance), without the need to introduce expensive isotope tracers into the patient. Isotope effects in metabolism are mostly caused by enzymatic reactions that preferentially consume substrates containing either the light or the heavy isotope (isotopologues) and therefore, the natural isotope abundance in metabolites depends on metabolic fluxes and source substrates^2^. For example, the natural ^13^C abundance in respired CO_2_ has been used to trace diet composition and substrate changes during exercise^3^,^4^. In cancer biology, the use of natural variations in Cu and S stable isotopes in hepatocellular carcinoma has been attempted recently^5^. But to our knowledge, no study has looked at alterations of natural isotope abundance in breast cancer. Due to changes in primary C and N metabolism such as increased glycolysis, glutaminolysis and nucleotide synthesis^6^, important changes in ^13^C and ^15^N natural abundance can be anticipated.

To address this question, we examined the isotopic signature of intact breast cancer biopsies (mostly from invasive ductal carcinoma, IDC) and cultured breast cancer cell lines (Supplementary Tables S1 and S2) using elemental analysis coupled to isotope ratio mass spectrometry (EA-IRMS). This technique has been recently shown to be applicable to the biochemical analysis of cancerous cell lines^7^.

## Results

### Isotope signature of breast cancer biopsies

**Figure 1:**
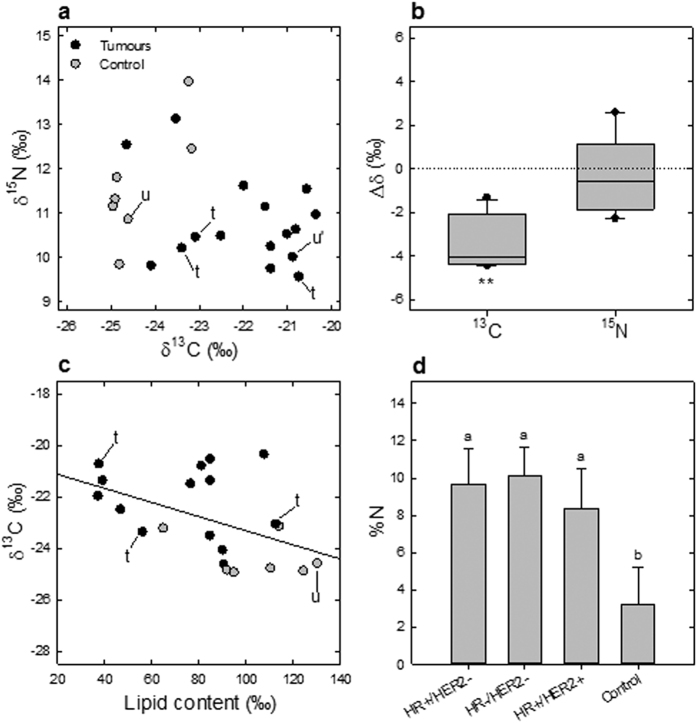
Breast cancer biopsies are naturally ^13^C-enriched and nitrogen-rich. (**a**) The natural abundance in ^13^C (δ^13^C, vs. V-PDB) and ^15^N (δ^15^N, vs. air N_2_) differentiates control from tumor patient tissue samples (n = 23). One sample pair of healthy (adjacent) and cancerous tissues not characterized for receptors (u and u’, respectively) and three triple negative tumor samples (t) are also shown. (**b**) Taken as a whole, the change in the ^13^C-abundance (Δδ) in cancerous tissues in tumor/adjacent tissue pairs was significant (*P* < 0.01, n = 5). **(c)** ^13^C-abundance (Δδ) was inversely correlated with the total lipid content (r^2^ = 0.48, *P* < 0.03). (**d**) Regardless of receptor expression, cancerous tissues had a higher elemental content in N (*P* < 0.05). In **(a**,**c**), each datum is the average of 3 sub-samples. In (**d**), lower case letters stand for statistical classes, *P* < 0.05.

Natural ^13^C and ^15^N abundance was found to discriminate normal and cancerous biopsies, the latter being significantly ^13^C-enriched by ~4‰ and tended to be (though not significantly) ^15^N-depleted (Fig. 1a,b). Triple negative tissues (ER- PR- HER2-) tended to be relatively ^15^N-depleted (Fig. 1a and Supplementary Table S1). The observed isotopic difference between cancerous and non-cancerous tissues can have been caused by either variation in chemical composition or isotope fractionations in metabolism.

First, there were alterations in total lipid content, which correlated to the ^13^C-abundance (Fig. 1c and Supplementary Figure S1). In fact, lipids are typically ^13^C-depleted components of human tissues^8^ and a systematic ^13^C-depletion has been observed in adipose tissue^9^. Here, cancerous tissues are on average less lipid-rich, reflecting the lower abundance of adipose cells in breast IDC^10^,^11^. Cancerous tissues were also enriched in nitrogenous compounds, as shown by the higher N elemental content (Fig. 1d). This effect is due to either a lower cellular N turnover rate or a higher content in protein and total amino acids including arginine (Supplementary Figure S2). Second, metabolic fluxes were modified thereby modifying the net exchange with the extracellular fluid and thus the overall (apparent) cellular isotope fractionation (see below). It is highly unlikely that the isotopic difference between biopsies was due to a confounding effect of nutritional preferences amongst patients (such as the proportion of animal proteins or sugars from C_4_-plants)^12^,^13^,^14^ because the comparison of paired normal and cancerous biopsy samples from the same patient still shows a significant difference for ^13^C (Fig. 1b). ^13^C and ^15^N abundance were also corrected so as to express them relative to a common elemental source (see Methods below). Moreover, female patients from which biopsies originated had the same geographical origin (western France) and thus important differences between nutritional habits or general isotope composition of body matter are improbable^15^.

### Metabolic alterations in breast cancer biopsies

Targeted metabolomics analyses by GC-MS were then carried out to identify major metabolic alterations. Normal tissues were clearly differentiated from cancerous tissues in both unsupervised (PCA) and supervised (orthogonal partial least square-discriminant analysis, OPLS-DA) analysis (Supplementary Figures S3 and S4), with ethanolamine, methionine and arginine having the highest weight in such a differentiation (Supplementary Figures S3 and S4). Other discriminating metabolites included carbohydrates (such as fructose) and putrescine, and these metabolites have also been found to be significant elsewhere^16^,^17^. While the effect on free ethanolamine reflected the general reorchestration of lipid metabolism^18^, the simultaneous effect on arginine and putrescine strongly suggests perturbations in arginine metabolism (urea cycle), thereby causing changes in N excretion and thus, by mass balance, in ^15^N-abundance and N elemental content.

### Isotopic signature of cell lines

Potentially, the isotopic signature in biopsies may have been influenced by non-controlled epidemiological and clinical factors (e.g., patient age, pharmacological treatments, etc.). Therefore, we also examined different cell lines cultured under isotopically-controlled conditions: one immortalized, non-cancerous cell line (MCF10A) and six breast cancer cell lines (ZR75-1, MCF7, SKBR3, MDA-MB-231, MDA-MB-468 and Cal51, see Supplementary Table S2). The use of cultured cells further allowed us to carry out isotopic compound-specific analyses as well as targeted gene-expression, metabolomics and EA-IRMS analyses (see Methods) on the same samples.

**Figure 2:**
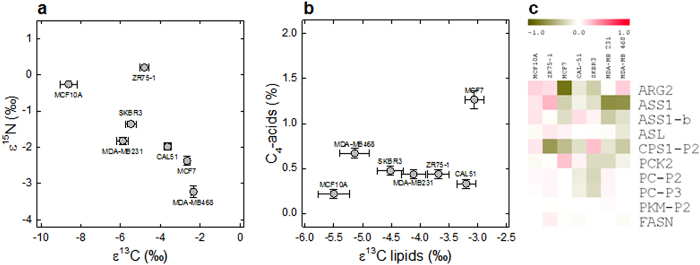
Cancerous cell lines are naturally ^13^C-enriched and ^15^N-depleted. When compared to source C and N used for growth, the natural isotopic enrichment (ε^13^C and ε^15^N) differentiates the control immortalized line (MCF10A) and the breast tumor ascites-derived line (ZR75-1) from other adenocarcinoma lines (**a**). The isotope enrichment in lipids parallels that in total organic matter, and cancerous lines contain more (^13^C-enriched) C_4_-acids (**b**). Despite some variations, transcript quantitation of key enzymes shows a generally lower expression in cancer cells compared to MCF10A cells of enzymes of the urea cycle, lipid synthesis or the anaplerotic pathway (gene abbreviations in Supplementary Table S5), suggesting that metabolite excretion in addition to biosynthesis is involved in the isotopic differences (**c**).

With the exception of ZR75-1 (non-pleural origin, ER+), all other breast cancer cell lines were ^15^N-depleted (Fig. 2a). The ^15^N-abundance in DNA, proteins and total soluble metabolites paralleled that in total organic matter (Supplementary Figure S5). The ^13^C-abundance in non-cancerous cells was found to be lower than in cancerous cells (Fig. 2a). It did not correlate to the lipid content but rather to the ^13^C-abundance in lipids and loosely, to the content in C_4_ organic acids (Fig. 2b) which are known to be ^13^C-enriched^19^.

### Primary metabolism in cell lines

**Figure 3:**
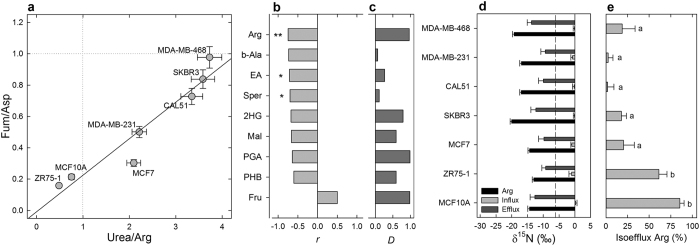
Cancerous cell lines excrete less ^15^N-depleted arginine (Arg). The role of Arg metabolism (urea cycle) is further demonstrated by the differences in ratios of endometabolites since Arg appears to be more retained relative to urea in cancerous cell lines (**a**). The extracellular-to-intracellular relative difference (*D*) in Arg was found to (i) be the best *D* value that anti-correlates to δ^15^N (**b**) and (ii) have the largest average *D* value across cell lines (**c**). In all cases, the δ^15^N value of the influx (substrate consumption) was close to 0‰ while the efflux (excreted N) was ^15^N-depleted and Arg was even more ^15^N-depleted in cancerous cell lines by up to ≈7‰ (**d**). Therefore, the relative isotopic efflux represented by Arg excretion (i.e., the quotient of Arg isoefflux to total isoefflux) was large in ZR75-1 and MCF10A and low in other cell lines (**e**). Asterisks: significant differences between cell lines (*P < 0.05; **P < 0.01); dashed line: δ^15^N of source Gln (**d**); lower case letters stand for statistical classes (*P* < 0.05) (**e**). EA, ethanolamine, 2HG, 2-hydroxyglutarate; PGA, phosphoglycerate; PHB, parahydroxybenzoate.

The endometabolome (i.e., intracellular metabolome) was analyzed to gain information on primary C and N metabolism (summarized in Supplementary Figure S6a). Some metabolites such as arginine, urea and glucose were significantly different between cancer and normal cell lines. Close correlations or anti-correlations were obtained between ^13^C- or ^15^N-abundance and key metabolites or metabolic ratios such as urea/arginine and fumarate/aspartate (Fig. 3a). As such, cancer cell lines could be differentiated from the MCF10A non-cancerous cells using these ratios (Fig. 3a).

The present results on both metabolome and isotopes suggest that three metabolic pathways are at the origin of the isotopic differences between cell lines: the urea cycle (arginine metabolism), the anaplerotic carbon fixation, and lipid synthesis. Transcript levels of a selection of genes encoding key enzymes of these pathways was examined by qPCR and in most cases, these genes were less expressed in cancer cell lines (Fig. 2c). Thus, rather than substantial changes in enzyme abundance, the alteration of isotope abundance was caused by changes in metabolic fluxes and/or net exchange of metabolites (absorption, excretion and accumulation) with the extracellular medium.

### Isotopic efflux in cell lines

We then examined the metabolic composition of the extracellular medium (exometabolome, Supplementary Figure S6b) to determine the preferential distribution (extracellular vs. intracellular) of metabolites and compute isotopic fluxes (isofluxes). The extracellular-to-intracellular difference and the ^15^N-abundance were found to be best anti-correlated with arginine, β-alanine, ethanolamine and spermidine (Supplementary Figure S6c,d). Amongst these, arginine had the highest value of the extracellular-to-intracellular difference (Fig. 3b,c) and was the most significantly different between cancer and normal cell lines (*P* < 0.005) and thus, appeared to be the best candidate to explain the ^15^N-depletion (Fig. 3d). There was little difference in the ^15^N-abundance of arginine itself amongst cell lines, although the non-cancerous (MCF10A) and ER lines (ZR75-1 and MCF7) synthesized ^15^N-enriched arginine (by a few ‰). Still, in all cases, arginine was much more ^15^N-depleted than the isotopic influx (isotope composition of substrates consumed by cells from supplied culture medium) or extracellular urea (Fig. 3d). As a result, arginine efflux from cells represented a very substantial part of the isotopic efflux to the extracellular medium in both ZR75-1 and MCF10A lines but was modest in others (Fig. 3e). That is, cancerous lines were naturally ^15^N-depleted (Fig. 3b) because of the proportionally lower excretion of ^15^N-depleted arginine. The higher metabolic ratio urea-to-arginine then comes as no surprise: it reflected the prevalence of arginine metabolism via the urea cycle (Fig. 3a).

## Discussion

**Figure 4:**
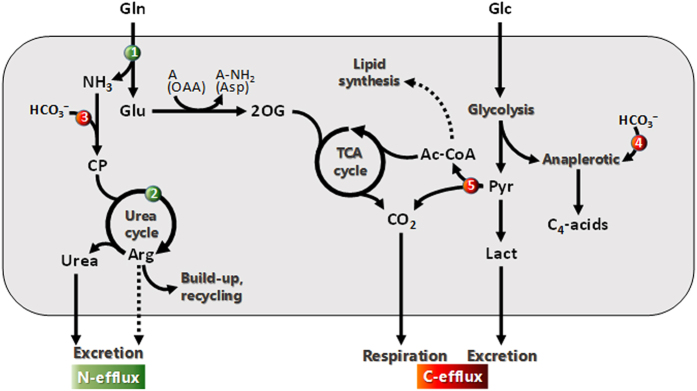
Major metabolic pathways are responsible for changes in the natural ^13^C and ^15^N abundance in cancerous cultured cells. Glutamine (Gln) is a major N and C source from which N is removed via hydrolysis (glutaminase ❶) and the urea cycle (❷). These reactions are both fractionating against ^15^N thereby yielding ^15^N-depleted urea and arginine (Arg). Therefore, build-up and recycling rather than excretion of Arg (dotted arrow) tend to deplete cancer cells of ^15^N. Cells are also ^13^C-enriched due to the fixation of bicarbonate by carbamoyl-phosphate (CP) synthesis (to feed the urea cycle ❸) and the anaplerotic pathway (❹), as well as a lower ^13^C content in non-structural lipids (dotted arrow). Lipids that are ^13^C-depleted come from the natural ^13^C-depletion in acetyl-CoA (Ac-CoA) (inherited from naturally ^13^C-depleted C-atom positions in glucose) and the isotope effect of pyruvate dehydrogenase^45^,^46^ (❺). Amine acceptors are denoted as ‘A’ and oxaloacetate (OAA) converted to aspartate (Asp) is provided as an example. 2OG, 2-oxoglutarate; Pyr, pyruvate; Lact, lactate. The potential contribution of respiration (CO_2_ loss) and lactate excretion to the natural ^13^C-abundance is described in Supplementary Table S4.

We showed isotopic differences between healthy and cancer tissues or cells, and provided evidence that these differences are related to urea, anaplerotic C fixation and/or lipid metabolism, and that the balance in nitrogen metabolism is modified in breast adenocarcinoma tissues or cells compared to normal breast tissues or cells thereby altering the natural ^15^N-abundance (Fig. 4). The consumption of glutamine (glutaminolysis) is a major pathway in cancerous cells^20^ that involves deamidation by glutaminase, thereby producing ammonia which is partly recycled and excreted in the form of urea. Here, source glutamine is naturally ^15^N-depleted (δ^15^N = −6.5‰) and ^14^N/^15^N isotope effects in both deamidation and the urea cycle^21^,^22^,^23^ yield ^15^N-depleted arginine. The efflux (excretion) of arginine does not counterbalance the N influx and this imbalance is compensated for by intracellular build-up and recycling into polyamines and β-alanine (Fig. 4) thereby causing a general ^15^N-depletion of organic matter. In addition to acid metabolism (C_4_-acids and lactate, Fig. 2b, Supplementary Table S3), the urea cycle incorporates bicarbonate (naturally ^13^C-enriched)^24^ via the action of carbamoyl phosphate synthase and thus, arginine build-up contributes to the enrichment of ^13^C in cells. This appears indeed to be the best explanation considering known isotope effects in metabolism^6^,^21^,^25^,^26^,^27^,^28^,^29^ (Supplementary Table S4). In fact, the natural ^13^C-enrichment cannot come from a change in total lipid content (as in tumors) since lipid content variation was small (Supplementary Figure S7) and furthermore, the isotope composition in lipids followed that in total organic matter (Fig. 2b).

Quite noticeably, the isotopic pattern seen in tumor biopsies was similar to that found in cell lines, that is, with a tendency to be ^15^N-depleted and ^13^C-enriched (Fig. 1a). This pattern is unlikely to come from an effect of nutrition, but rather explained by the specific primary C and N metabolism in tumor tissues, as explained above (Fig. 1). The metabolic origin of the ^13^C-enrichment was different to that in cultured cells (a decrease in total lipid content in tumors, Supplementary Figure S7 and refs 18,30). This is not surprising since cultured cell metabolism is different from that of cells *in situ*. By contrast, the mechanism explaining the lower ^15^N-abundance was similar: a net influx of ^15^N-depleted nitrogen associated with any metabolism. In cancerous cultured cells, there is an increased activity of the urea cycle and non-quantitative excretion of ^15^N-depleted arginine. In tumors *in situ*, it is believed that cells are arginine-dependent to sustain polyamine metabolism^31^, and the auxotrophy of tumors for arginine has been exploited in the development of anticancer therapies^32^. Therefore cells capture naturally ^15^N-depleted arginine from their microenvironment, thereby leading to a ^15^N-depletion of cellular organic matter. Such a mechanism agrees with the well-known increase in polyamine metabolism^33^ and the fact that urea and glutamine (glutaminase activity) are amongst the best urinary and tumor biomarkers for breast cancer, respectively^34^,^35^. Also, differential expression of arginase, a key enzyme of the urea cycle, has been reported in breast cancer cell lines^36^,^37^.

The natural abundance in ^13^C and ^15^N thus reflects primary metabolism of cancer cells and thus, offers potential opportunities for development of biomarkers for clinical research. Consistently, healthy and cancerous samples were found to fall in corresponding regions of the isotopic landscape (Fig. 1a), and normal and cancerous cell lines could be differentiated (Fig. 2a). The ability of natural ^13^C- or ^15^N-abundance to further differentiate tumor grade and severity remains to be explored. In addition, there is a potential use the natural isotope abundance to distinguish between different breast cancer subtypes, as they have been shown to have different metabolic phenotypes^38^. Also, isotopic measurements would be best adapted to clinical settings if measured in plasma metabolites rather than in biopsies and thus, future work on both the accuracy and applicability of such plasma isotopic markers is required. For example, ^15^N natural abundance in plasma arginine could be exploited as a marker provided the metabolic activity of the tumor has a detectable effect on the total circulating arginine pool. That isotopic signature of tumorous breast tissues is due to alterations in major pathways of primary metabolism highlights the potential relevance of our findings to other cancer types which also display changes in metabolic fluxes.

## Methods

### Human Tissues

Fresh human mammary samples were obtained from chemotherapy naive patients with invasive carcinoma after surgical resection at the Institut de Cancérologie de l’Ouest – René Gauducheau, Nantes, France. Informed consent was obtained from patients to use their surgical specimens and clinicopathological data for research purposes, as required by the French Committee for the Protection of Human Subjects (CCPPRB). Ouest IV – Nantes CCPPRB approved use of tumor tissues for this study (6 May 2013: no. 357/2013). The collection of tumors was approved by the French Minister of higher education and research (no. AC-2008-141). This study did not require additional ethical approval. Amongst 17 breast cancer patients, 12 breast cancer samples and 5 pairs of breast cancer and adjacent non-cancerous tissues were obtained. One normal breast sample was obtained from reduction mammoplasty. For tumors, hormonal receptors, histological type and grade information were available, except that a paired of tissue #22/#19 (Supplementary Table S1, u and u’ in Fig. 1a) did not have receptor status available.

### Cultured cells

Six human breast cancer cell lines and one non-cancerous cell line (Supplementary Table S2) were used and grown as monolayer cultures (10^7^ cells/75  m^2^ flask). The cancer cells were grown in no-glucose no-glutamine DMEM supplemented with 10% FBS and 1% 10,000 units/ml penicillin, 10,000 units/ml streptomycin at 37 °C in a humidified atmosphere of 5% CO_2_. The non-cancerous cell line (MCF10A) was grown in DMEM/F-12 (no-glucose no-glutamine) supplemented with 5% horse serum, 0.01% EGF (Epidermal Growth Factor), 0.005% cholera toxin, 0.1% insulin, 0.005% hydrocortisone, and 1% HEPES. L-glutamine and D-glucose (4 and 25 mM, respectively) were added separately to culture mediums to ensure these nutrients were isotopically identical for all cells. The same cells were grown several times to test the biological variability. As soon as cells were confluent (~10^7^ cells), cell counting and extraction were done using triplicate cell culture flasks. One flask was used for cell counting, after harvesting with trypsin, using trypan blue staining and microscopic hemocytometry. For other analyses carried out on the other flasks, cells were then prepared as an aqueous (isotopes) or methanolic (metabolomics) extract without using trypsin as explained below.

### Extraction and separation of studied fractions

#### Human tissues

Human fresh tissues were lyophilised. Lipids were extracted from the dried human tissues (10 mg) with hexane (1 mL). After centrifugation (4000 rpm, 15 min, 4 °C), the pellet was kept for metabolomics and the lipids were recovered by evaporation of hexane under a stream of N_2_ gas at 40 °C.

#### Cultured cells

After culture, cells were washed twice with PBS, and 3 mL of cold water was added to the flask. Cells were detached from the flask using a cell scraper. The lysed cell preparation was kept at −20 °C for 48 h and then lyophilized. Different fractions (lipids, total soluble fraction (TSF), protein and DNA) were extracted and separated from the dry cell material. To obtain a lipid fraction, lyophilized cells (40 mg) were ground and extracted twice under agitation with 2 mL cyclohexane for 5 min at 20 °C. After centrifugation (4000 rpm, 5 min, 20 °C), the lipid fraction (4 mL of cyclohexane) was dried under a stream of N_2_ gas at 40 °C and the dry residue stored at −20 °C until required. To obtain a total soluble fraction, the pellet (insoluble in cyclohexane) obtained after centrifugation were ground and extracted twice with 2 mL methanol/water (80:20 v/v) for 5 min at 20 °C. After centrifugation (4000 rpm, 5 min, 20 °C), the total soluble fraction was dried under a stream of N_2_ gas at 40 °C and the dry residue stored at −20 °C until required. The pellet (insoluble in methanol/water) consisted of a protein fraction.

For DNA purification, lyophilised cells (15 mg) were reconstituted with 1 mL water and extracted with an equal volume of phenol/chloroform in an ultrasound bath for 30 min. After centrifugation (4000 rpm, 5 min, 4 °C), the aqueous phase was filtered to remove proteins and the DNA was precipitated with an equal volume of isopropanol (1 mL). After gentle agitation, the DNA fragments were pelleted by centrifugation (4000 rpm, 5 min, 4 °C), washed twice with ethanol (70%) to remove impurities, and dried under a stream of N_2_ gas at 40 °C.

### ^13^C and ^15^N abundance by elemental-analysis coupled to isotope ratio mass spectrometry (EA-IRMS)

Subcellular fractions (see above) were lyophilized for EA-IRMS analysis, in order to detect their ^13^C and ^15^N isotopes. From each sample ~0.7 mg was weighed with 10^−5^ g precision balance (Ohaus Discovery DV215CD, Pine Brook, New Jersey, USA) into each of three tin capsules (solids “light” 5 × 9 mm, Thermo Fisher Scientific, www.thermo.com) for isotope analysis. EA-IRMS analyses were performed as previously described^7^. The ^13^C and ^15^N abundance was first expressed as δ values (δ^13^C and δ^15^N, in ‰) as δ = *R*/*R*_st_ − 1 where *R* is the heavy-to-light isotope ratio and *R*_st_ stands for the isotope ratio in the international reference (V-PDB for ^13^C and air N_2_ for ^15^N).

The isotope abundance in sample fractions was expressed relative to that in total organic matter using the enrichment (in ‰) ε = δ_f_ − δ_tom_ where δ_f_ is the δ value in the fraction of interest (lipids, proteins, etc.) and δ_tom_ the (non-corrected) δ value in total organic matter. The enrichment ε is then positive if the sample fraction is enriched in the heavy isotope and negative if it is depleted. In this way, ε nearly equals the opposite of the isotope fractionation Δ (which is given by [δ_tom_ − δ_f_]/[δ_f_ + 1] ≈ δ_tom_ − δ_f_). For total organic matter in Figs 1 and 2 and Supplementary Figure S3, δ values were corrected to allow comparisons between patients (that may have had different nutritional preferences) and between cell lines (that had slightly different culture media), as follows:

In cultured cells, it was corrected for the δ value of the elemental C and N source using the classical relationships accounting for isotope fractionation: δ_corr_ = (δ_av_ − Δ)/(Δ + 1) where Δ = (δ_srce_ − δ)/(δ + 1), δ_srce_ is the δ value of the elemental source and δ_av_ is δ value used as the reference (here, the average of δ_srce_ values was used as the reference value; note that any change in this value would not change the pattern seen here, it would only change the absolute scale of δ_corr_ values). δ_srce_ was the δ^15^N and δ^13^C of source amino acids and sugars, respectively, in intact culture medium. When expressed as a fractionation (Fig. 2a), the isotope composition in total organic matter of cells is given as ε = δ − δ_srce_. That way, ε values are positive when it is enriched in the heavy isotope and negative when it is depleted, as for ε in individual cell fractions (see above).

For biopsies, δ_srce_ could not, quite obviously, be known because food composition of patients was not controlled. We therefore had to use a correction that could be calculated independently of food composition. The major influence of nutrition on elemental composition and isotopes is the protein/fat ratio of ingested food^39^, with fat-rich and protein-poor food causing a general increase in %C (and decrease in %N) and a general decrease in both δ^15^N and δ^13^C in body tissues. Therefore, we renormalized the δ values in total organic matter by taking into account the %C and %N using δ_corr_ = δ + (1 − *f* )τ where *f* is the elemental composition relative to the average and τ is the apparent isotope fractionation between the whole population δ value (average isotope composition of human tissues in European western (French) population (δ_a_), −22‰ and 9‰ for ^13^C and ^15^N, respectively) and the average of our samples (δ_m_): τ = δ_a_ − δ_m_. For example, in this way, protein-rich samples (lean patients) that are naturally impoverished in elemental C and richer in ^13^C will have a negative correction for δ^13^C. Of course, such a correction was not ideal because the knowledge of the average isotope composition of ingested food would have been better. In practice, this correction was minimal (on average, 0.02‰ for ^13^C and 0.5‰ for ^15^N) and did not cause a substantial bias in our data.

### ^15^N-abundance and amino acid content using gas chromatography - combustion - isotope ratio mass spectrometry (GC-C-IRMS)

When cells were confluent, the culture medium was kept (10 mL from ~10^8^ cells), and cells (~10^8^) were washed with PBS and then lysed in 36 mL of 80% methanol. After homogenization, methanolic extracts were dried under a stream of N_2_ gas at 40 °C. Amino acids were prepared as their *N*-pivaloyl, *O*-isopropyl esters for GC-C-IRMS analysis, and used for both quantification (μmol) and ^15^N values (‰) as previously described^40^.

### ^**13**^C-abundance in metabolites using liquid chromatography coupled to chemical oxidation and isotope ratio mass spectrometry (LC-co-IRMS)

For LC-co-IRMS analysis, ~10^7^ cells were used in 12 mL of 80% methanol. LC-co-IRMS analysis was performed as previously described, using water as a mobile phase and a RS Pak KC-811 column (Shodex)^41^. δ^13^C values were corrected using pure standard solutions at different concentrations. In practice, such corrections due to non-linearity were very small, about 0.2‰.

### GC-MS metabolomics

#### Human biopsies

If material remained after lyophilization and lipid extraction, an additional ~10–15 mg of tissue was ground and dissolved in a standard solution of DL-2-Aminobutyric acid (0.2 mM, 950 μL) and methanol/water (950 μL, 80:20 v/v) was added to extract the soluble metabolites. After centrifugation (14000 rpm, 15 min, 4 °C), the soluble fraction was mixed with standard solution of ribitol (2 mM, 95 μL). The solution was centrifuged again and three aliquots of 200 μL were dried using a speed-vac before derivatization and GC-MS analysis as described previously^41^,^42^.

#### Cultured cells and extracellular medium

About 5 × 10^7^ cells were dissolved in a solution of methanol/water (7 mL, 60:40 v/v) containing 500 μL of a standard solution D,L-2-aminobutyric acid (0.2 mM). After centrifugation (14000 rpm, 15 min, 4 °C), the soluble fraction was mixed with another standard solution of ribitol (2 mM, 50 μL). The solution was centrifuged again and three aliquots of 1.4 mL, i.e. 20% of ~5 × 10^7^ cells, were dried using a speed-vac before derivatization and GC-MS analysis. For extracellular medium, the same standard solutions [D,L-2-aminobutyric acid (0.2 mM, 500 μL) and ribitol (2 mM, 50 μL) and methanol/water solution (500 μL, 60:40 v/v)] were added to 500 μL of media collected from the cultured cells. After a centrifugation, three aliquots of 300 μL were prepared for GC-MS analysis. All metabolomics data were normalized to the specific number of cells so that the exo- and endo-metabolome could be directly compared.

### RT-qPCR

RNA was isolated from cell lines and 500 ng RNA was reverse transcribed as previously described^43^. Quantitative PCR was done using the Maxima SYBR Green/ROX qPCR Master Mix (Life Technologies) and the MX4000 instrument (Stratagene, Basel, Switzerland), according to the manufacturer’s instructions. The endogenous housekeeping genes *RPLPO*, *HPRT1* and *ACTB1* were used for normalization. Relative quantification was carried out by using the ΔΔCt method. Primer sequences are listed in Supplementary Table S5.

### Statistics

Pair-wise comparisons were carried out using Student-Welsh tests, with a p-threshold value of 0.01 unless otherwise stated in text. Metabolomics were examined using unsupervised analysis with principal component analysis (PCA) or supervised analysis with orthogonal partial least square discriminant analysis (OPLS-DA), both carried out with Simca (Umetrics). Heat maps and hierarchical clustering (HCL) were done using the open source software MeV^44^. The HCL was carried out using the cosine correlation as a measure of proximity.

## Additional Information

**How to cite this article**: Tea, I. *et al*.^13^C and ^15^N natural isotope abundance reflects breast cancer cell metabolism. *Sci. Rep.* **6**, 34251; doi: 10.1038/srep34251 (2016).

## Supplementary Material

Supplementary Information
