# Supplementary material for: 13C and 15N natural isotope abundance reflects breast cancer cell metabolism
Source: Sci Rep. 2016 Sep 28;6:34251. doi: 10.1038/srep34251 (PMC5039687; doi:10.1038/srep34251)
Supplement: Supplementary Information [file srep34251-s1.doc]

**Supporting information**

**^13^C and ^15^N natural isotope abundance reflects breast cancer cell metabolism**

Illa Tea^a,b,1^, Estelle Martineau^a,c^, Ingrid Antheaume^a^, Julie Lalande^a^, Caroline Mauve^d^, Francoise Gilard^d^, Sophie Barillé-Nion^e^, Anneke C. Blackburn^b^ and Guillaume Tcherkez^f^

^a^ EBSI team, CEISAM, University of Nantes-CNRS UMR 6230, 2 rue de la Houssinière BP 92208, F-44322 Nantes, France.

^b^ Cancer Metabolism and Genetics Group, The John Curtin School of Medical Research, The Australian National University, Canberra 2601 ACT, Australia.

^c^ Spectromaitrise, CAPACITÉS SAS, 26 Bd Vincent Gâche, 44200 Nantes, France

^d^ Plateforme Métabolisme-Métabolome, Bâtiment 630 IPS2, Université Paris-Sud, 91405 Orsay cedex, France.

^e^ CRCNA, UMR INSERM U892 / CNRS 6299 / Université de Nantes, Team 8 « Cell survival and tumor escape in breast cancers », Institut de Recherche en Santé de l’Université de Nantes, 8 quai Moncousu, BP 70721, 44 007 Nantes cedex 1, France

^f^ Research School of Biology, The Australian National University, Canberra 2601 ACT, Australia.

^1^To whom correspondence should be addressed: illa.tea@anu.edu.au

**This file includes:**

• Supplementary Figures 1 to 7

• Supplementary Tables 1 to 5

**Supplementary figures**

**Fig. S1: The natural ^13^C-enrichment in lipids inversely correlates tightly to ^13^C-abundance in organic matter (ε^13^C lipids, expressed relative to total organic matter) with** r^2^ = 0.93 (*y* = 0.86*x* – 22.06). Black discs, healthy tissues; grey discs, cancerous tissues. Samples of healthy tissue from plastic surgery (a) and from not characterized for receptors (u) and two triple negative tumor samples (t) are labelled with letters. Note that data on lipids were not available for all samples because the initial amount of tissue sample was insufficient for all biochemical analyses.

**Fig. S2:** **Cancerous tissues are richer in arginine (Arg):** Arg content of tumor tissues relative to control tissues, redrawn from source data of Supplementary Figure 1 above. (*, *P*<0.08 ; **, *P*<0.01 ; dotted line : reference line at 1)

**Fig. S3:** **Unsupervised analysis** **of the metabolome of biopsies differentiates tumors and control samples. (a)** Heat map representation and hierarchical clustering showing 80 metabolites. (**b**) Significant metabolites in univariate analysis (*P*<0.01). **(c)** The scatter plot of the principal components analysis (PCA) also shows a separation of tumors (black) and healthy (grey) samples. The contribution of principal component 1 and 2 was 38 and 22%, respectively. Two metabolites of Arg metabolism were found to be significant: Arg and putrescine (one way anova, *P*<0.01).

**Fig. S4: Supervised multivariate analysis (OPLS-DA) of tissue metabolome differentiates tumors** (grey) and control (black) samples (**a**). The 20% best metabolites (highest loading values and variable importance for the projection) include ethanolamine and Arg, both being more abundant in tumors (**b**) (loading is expressed as % of the maximum value). The OPLS-DA was associated with a satisfactory predictive power (r²=0.66).

**Fig. S5: Cellular biochemical fractions are not isotopically very distinct from total organic matter:** the ^15^N-enrichment **(ε^15^N, relative to total organic matter)** of DNA, proteins, and total soluble fraction (TSF) are all less than 1.5‰-different from total organic matter (left). Total free amino acids (ε^15^N average, cyan, right) tends to be more variable but appear to be isotopically more distinct from total organic matter in ZR75-1 and MCF10A cell lines.

**Fig. S6:** **Intracellular-to-extracellular difference best correlates to δ^15^N for arginine** (Arg)**.** Endometabolome **(a)** and exometabolome **(b)** analysis shows differences between cell lines, such as a higher content in intracellular glucose in both MCF-10A and ZR-75-1. Endo- and exo-metabolome are then combined to calculate an intra vs extracellular difference **(c)** such that negative values represent enrichment in the extracellular medium and are thus instrumental in detecting potential metabolite efflux. Despite variation between cell lines, some metabolites have a consistent pattern, such as metabolites hardly liberated into the medium and retained within cells: e.g., putrescine, glucose-6-phosphate and 3-phosphoglycerate. The Pearson correlation coefficient r with the natural abundance in ^15^N is the largest in absolute value (most negative) with Arg, suggesting that Arg efflux is at the origin of the ^15^N signature (**d**, in red). The purple vertical dashed line represents the average of negative r values across metabolites. In **a** and **b**, data have been rescaled to the maximum value for each metabolite (max-ranging). In **c**, positive (respectively negative) values have been rescaled with respect to the maximum (respectively minimum) value across all metabolites.

**Fig. S7**: **The elemental content in total lipids (in** ‰, i.e., in mg C g^-1^ C**) in tissues (tumors and control) and cultured cells show differences: tumors tend to be lipid-depleted compared to control tissues (*P*<0.07), and cells are much less lipid-rich than biopsies. Thus in contrast to biopsies (See Fig. 1, main text), lipids are unlikely to explain changes in ^13^C-abundance between cultured cells lines.**

**Supplementary tables**

**Table S1: Histopathological properties of biopsied samples**. HR, Hormonal receptors (oestrogen (ER) and progesterone receptor (PR)); IDC, invasive ductal carcinoma; ACC, adenoid cystic carcinoma; ILC, invasive lobular carcinoma; ND, not determined; n/a, not applicable

|  | |  |  |  |  |  |
| --- | --- | --- | --- | --- | --- | --- |
| **Sample ID** | | **Hormonal receptors** | **HER2** | **Histological type** | **grade** |  |
| **Cancerous tissues (unpaired) :** | | | | | |  |
| #1 | | HR+ | HER2- | IDC | 3 |  |
| #2 | | HR+ | HER2- | IDC | 2 |  |
| #3 | | HR+ | HER2+ | IDC | 3 |  |
| #4 | | HR+ | HER2- | ND |  |  |
| #5 | | ER+ | HER2- | IDC | 2 |  |
| #6 | | HR+ | HER2- | IDC | 2 |  |
| #7 | | HR+ | HER2- | IDC | 2 |  |
| #8 | | HR+ | HER2- | IDC | 3 |  |
| #9 | | HR+ | HER2+ | IDC | 3 |  |
| #10 | | HR- | HER2- | ACC |  |  |
| #11 | | ND | ND | n/a |  |  |
| #12 | | HR+ | HER2- | ND |  |  |
| **Paired samples :** | | | | | | |
| #13/#14 | | HR+ | HER2- | ILC | 3 |  |
| #15/#16 | | HR- | HER2- | IDC | 3 |  |
| #17/#18 | | HR- | HER2- | IDC | 3 |  |
| #22/#19 | | ND | ND | n/a |  |  |
| #23/#20 | | HR+ | HER2- | IDC | 1 |  |
|  | |  |  |  |  |  |
| **Normal tissue (reduction mammoplasty) :** | | | | | |  |
| #21 | Not Applicable | | | | |  |
|  | |  |  |  |  |  |

**Table S2: Characteristics of breast epithelial cell lines** used in the present study. All patients were female. NA, not amplified; n/a, not applicable. ER: estrogen receptor; PR: progesterone receptor.

| **Cell Line Name** | | **Breast cancer subtype** | **Patient Age** | | **Histology** | | | | **Source** | | **ER** | **PR** | **HER2** |
| --- | --- | --- | --- | --- | --- | --- | --- | --- | --- | --- | --- | --- | --- |
| MCF7 | | luminal | 69 | | Adenocarcinoma-mammary gland; breast | | | | Pleural effusion | | + | - | - |
| MDA-MB-231 | | mesenchymal | 51 | | Adenocarcinoma-mammary gland; breast; epithelial | | | | Pleural effusion | | - | - | NA |
| SKBR3 | | luminal | 43 | | Adenocarcinoma-mammary gland; breast; epithelial | | | | Pleural effusion | | - | - | + |
| CAL51 | | mesenchymal | 45 | | Adenocarcinoma-mammary gland; breast; epithelial | | | | Pleural effusion | | - | - | NA |
| MDA-MB-468 | | basal | 51 | | Adenocarcinoma-mammary gland; breast; epithelial | | | | Pleural effusion | | - | - | NA |
| ZR75-1 | | luminal | 63 | | Ductal carcinoma-mammary gland:breast/duct. Non pleural | | | | Ascites | | + | - | NA |
| MCF10A | | n/a | 36 | | Mammary gland; breast; fibrocystic disease: (non-cancerous) | | | | Breast | |  |  |  |
|  | |  |  | |  | | | |  | |  |  |  |
|  |  | | |  | |  |  |  | |  | | | |

**Table S3: ^13^C-enrichment (ε^13^C, relative to total organic matter) in metabolites** (lactate, organic acids [citrate, malate, pyruvate, not resolvable by LC], glucose and glutamine) determined using liquid chromatography coupled to chemical oxidation and isotope ratio mass spectrometry (LC-co-IRMS) shows that despite visible variation (order of magnitude of SD), ZR75-1 and MCF10A have ^13^C-depleted lactate and ^13^C-enriched glucose compared to other cell lines. As a result, the apparent fractionation during glucose-to-lactate conversion (denoted as Δ) was against ^13^C (> 0) while it was against ^12^C (< 0) in other lines. Significantly different classes of values (one way anova p<0.05) are in bold. The average SD over all lines is shown.

|  | **^13^C-enrichment (ε^13^C), ‰** | | | | | | **Δ, ‰** |
| --- | --- | --- | --- | --- | --- | --- | --- |
|  | | Lactate | | Organic acids. | Glucose | Glutamine |
| **MCF7** | | | 0.34 | -2.72 | -7.33 | 3.49 | -7.7 |
| **MDA-MB-231** | | | -0.72 | 3.46 | -5.00 | 4.05 | -4.3 |
| **SKBR3** | | | 4.69 | 0.87 | -4.54 | 5.06 | -9.3 |
| **CAL51** | | | -2.49 | 1.18 | -11.05 | 5.53 | -8.7 |
| **MDA-MB-468** | | | 2.22 | -1.47 | -3.33 | 2.04 | -5.6 |
| **ZR75-1** | | | **-9.91** | 1.33 | **-0.21** | 4.28 | **9.7** |
| **MCF10A** | | | **-7.72** | 5.10 | **-0.71** | 7.09 | **7.0** |
|  | | |  |  |  |  |  |
| Average SD | | | 0.94 | 3.52 | 0.28 | 1.12 | 0.5 |

**Table S4: Analytical examination of the possible origins of the ^13^C-enrichment in cultured breast cancer cells.** In summary, the two likely metabolic events that enrich in ^13^C cancerous cell lines appear to be the fixation of bicarbonate and lactate metabolism.

| **Possible origin of the observed ^13^C-enrichment** | **Possible occurrence and pieces of evidence** | **Credibility** |
| --- | --- | --- |
| **Absorption of ^13^C** |  |  |
| Fractionation (isotope effect) in favour of ^13^C in substrate assimilation per se (here, the substrate is C-source mostly in the form of glucose or glutamine) | Fractionation related to transporters is highly unlikely since all transport phenomena (including across membranes or permeable media) fractionate very weakly, in favor of ^12^C. Glutamine hydrolysis fractionates against ^13^C (see Supplementary Table 3) but the difference between cells is insignificant. | Unlikely |
| Incorporation of ^13^C-enriched molecules (like bicarbonate) | This incorporation is likely, because both anaplerosis (oxaloacetate synthesis by the phospho*enol*pyruvate carboxylase, for example) and carbamoyl phosphate synthesis consume bicarbonate, which is 9‰-enriched compared with dissolved CO_2_ (isotope effect in hydration). | Likely |
| **Loss of ^12^C** |  |  |
| Excretion of ^13^C-depleted metabolites or non-quantitative excretion of ^13^C-enriched metabolites (like lactate) | Compound-specific isotopic analyses show that lactate was isotopically close to total organic matter, except in ZR75-1 and MCF10A lines where it was ^13^C-depleted (see Supplementary Table 3). There was an apparent fractionation in favor of ^13^C during the glucose-to-lactate conversion (fermentation) in all cell lines except for ZR75-1 and MCF-10A. The non-quantitative excretion of lactate into the medium could have thus contributed to enrich cancerous cells in ^13^C. | Likely |
| Liberation of ^12^CO_2_ by respiration | It is known that there is little isotope effect in CO_2_ production from source C in respiration unless substrate shifts occur, such as lipid remobilization (β-oxidation) which evolves ^13^C-depleted CO_2_. It is believed that cancerous cells rather produce fatty acids to match needs in membrane synthesis (cell division) and therefore, respired CO_2_ is probably moderately ^13^C-depleted. In addition, a significant fraction of the carbon source feeding the Krebs cycle is 2-oxoglutarate from glutaminolysis, and cellular glutamine was found to be isotopically similar in all cell lines (see Supplementary Table 3). | Unlikely |

**Table S5: Primer sequences for RT-qPCR**. Abbreviations are as follows: for ARG2, arginase; ASS1, arginosuccinate synthase; ASS1-b, ASS1 variant; ASL, argininosuccinate lyase ; CPS1-P2, carbamoyl-phosphate synthase 1; PCK2, phosphoenolpyruvate carboxykinase 2; PC-P2, phosphoenolpyruvate carboxylase 2; PC-P3, phosphoenolpyruvate carboxylase 3; PKM-P2, pyruvate kinase 2; FASN, fatty acid synthase.

| **Gene** | **Primer Sequence** | |  |  |  |
| --- | --- | --- | --- | --- | --- |
|  |  |  |  |  |  |
| ARG1 | Forward primerAGCATGAGCGCCAAGTCCAGA | | | |  |
|  | Reverse primerTCTCAAGCAGACCAGCCTTTCTCA | | | |  |
|  |  |  |  |  |  |
| ARG2 | Forward primerGCAGCCTCTCGCGTCTCCTCC | | | |  |
|  | Reverse primerGCGGGACCATGCTCCACTCCTT | | | |  |
|  |  |  |  |  |  |
| ASS1 | Forward primerCCAGTCCTGCTCTGCCGCCT | | | |  |
|  | Reverse primerAGGCGTGAGTTCCCGGCGTC | | | |  |
|  |  |  |  |  |  |
| ASS1Bis | Forward primerACCCCTGCCAGTCCTGCTCTG | | | |  |
|  | Reverse primerCCACGGAGCCTTTGCTGGACA | | | |  |
|  |  |  |  |  |  |
| ASL | Forward primerCAGACCCGGAGGACCGAAGC | | | |  |
|  | Reverse primerCTGCACCCACAAACCGGCCA | | | |  |
|  |  |  |  |  |  |
| PCK1 | Forward primerCCATTCAGCATGGGGCCGCT | | | |  |
|  | Reverse primerCACTGCTTCCAGGACGGGCG | | | |  |
|  |  |  |  |  |  |
| PCK2 | Forward primerCCAGGTGCCATGGCCGCATT | | | |  |
|  | Reverse primerCTACGGCATGATGGCCAGCCC | | | |  |
|  |  |  |  |  |  |
| FASN | Forward primerCGATGACCGTCGCTGGAAGGC | | | |  |
|  | Reverse primerTCGTGTGTGCCTGCTTGGGG | | | |  |
|  |  |  |  |  |  |
|  |  |  |  |  |  |
|  |  |  |  |  |  |
